# Evaluation of Drug—Drug Interactions in EGFR-Mutated Non-Small-Cell Lung Cancer Patients during Treatment with Tyrosine-Kinase Inhibitors

**DOI:** 10.3390/jpm11050424

**Published:** 2021-05-18

**Authors:** Mario Occhipinti, Marta Brambilla, Giulia Galli, Sara Manglaviti, Maristella Giammaruco, Arsela Prelaj, Roberto Ferrara, Alessandro De Toma, Claudia Proto, Teresa Beninato, Emma Zattarin, Giuseppe Lo Russo, Alain Jonathan Gelibter, Maurizio Simmaco, Robert Preissner, Marina Chiara Garassino, Filippo De Braud, Paolo Marchetti

**Affiliations:** 1 Thoracic Oncology Unit, Fondazione IRCCS Istituto Nazionale dei Tumori, Via Giacomo Venezian 1, 20133 Milan, Italygiulia.galli@istitutotumori.mi.it (G.G.); sara.manglaviti@istitutotumori.mi.it (S.M.); arsela.prelaj@istitutotumori.mi.it (A.P.); roberto.ferrara@istitutotumori.mi.it (R.F.); alessandro.detoma@istitutotumori.mi.it (A.D.T.); claudia.proto@istitutotumori.mi.it (C.P.); teresa.beninato@istitutotumori.mi.it (T.B.); emma.zattarin@istitutotumori.mi.it (E.Z.); giuseppe.lorusso@istitutotumori.mi.it (G.L.R.); marina.garassino@istitutotumori.mi.it (M.C.G.); filippo.debraud@istitutotumori.mi.it (F.D.B.); 2 Department of Experimental Medicine, Sapienza University of Rome, Viale Regina Elena, 324, 00161 Rome, Italy; 3 Medical Oncology Unit B, Policlinico Umberto I, Sapienza University of Rome, Viale del Policlinico, 155, 00161 Roma, Italy; maristella.giammaruco@uniroma1.it (M.G.); alain.gelibter@uniroma1.it (A.J.G.); paolo.marchetti@uniroma1.it (P.M.); 4 Department of Electronics, Information, and Bioengineering, Politecnico di Milano, Via Giuseppe Ponzio, 34, 20133 Milano, Italy; 5 Department of Neuroscience, Mental Health and Sensory Organs (NESMOS), Faculty of Medicine and Psychology, Sapienza University of Rome, Sant’Andrea University Hospital, Via di Grottatossa, 1035, 00189 Rome, Italy; maurizio.simmaco@uniroma1.it; 6 Department of Advanced Molecular Diagnostics, Sapienza University of Rome, Sant’Andrea Hospital, Via di Grottatossa, 1035, 00189 Rome, Italy; 7 Institute of Physiology and Science-IT, Charité–Universitätsmedizin Berlin, Corporate Member of Freie Universität Berlin, Humboldt-Universität zu Berlin, and Berlin Institute of Health, Charitéplatz 1, 10117 Berlin, Germany; robert.preissner@charite.de; 8 Knapp Center for Biomedical Discovery, University of Chicago Medicine & Biological Sciences, 900 E 57th St, Chicago, IL 60637, USA; 9 Department of Clinical and Molecular Medicine, Sapienza University of Rome, Via di Grottarossa, 1035, 00189 Rome, Italy

**Keywords:** drug–drug interactions (DDI), EGFR, tyrosine-kinase inhibitors (EGFR-TKIs), non-small-cell lung cancer (NSCLC), Drug-PIN^®^

## Abstract

(1) Background. The onset of a drug–drug interaction (DDI) may affect treatment efficacy and toxicity of advanced non-small-cell lung cancer (aNSCLC) patients during epidermal growth factor receptor (EGFR) tyrosine-kinase inhibitor (TKI) use. Here we present the use of Drug-PIN^®^ (Personalized Interactions Network) software to detect DDIs in aNSCLC patients undergoing EGFR-TKIs. (2) Methods. We enrolled patients with Stage IV aNSCLC already treated with or candidates to receive EGFR-TKIs, in any line; ECOG PS 0–2; taking at least one concomitant drug. Cancer treatments, concomitant drugs, and clinical and laboratory data were collected and inserted in Drug-PIN^®^. (3) Results. Ninety-two patients, median age of 68.5 years (range 43–89), were included. In total, 20 clinically relevant DDIs needing medical intervention in a total of 14 patients were identified; the 14 major DDIs were related to a high-grade interaction between TKIs and SSRIs, antipsychotics, antiepileptics, H2-receptor antagonist and calcium antagonists. A negative association between statin intake and PFS was identified (*p* = 0.02; HR 0.281, 95% CI 0.096–0.825). (4) Conclusions. This is the first retrospective study assessing the prevalence of DDIs, the clinical need for medical intervention and the impact of concomitant drugs on EGFR-TKIs survival in aNSCLC.

## 1. Introduction

A drug to drug interaction (DDI) is defined as “the pharmacological or clinical response to the administration of a drug combination, different from that anticipated from the known effects of the two agents when given alone” [1]. DDIs can be divided into two classes according to in vivo mechanism of interaction: pharmacokinetic (absorption, distribution, metabolism and excretion) and pharmacodynamic (receptor function, biological process and additive/opposed effect) [2]. DDIs can lead to critical clinical effects and the development of adverse drug reactions (ADRs). Moreover, they can reduce or increase the efficacy of therapies. DDIs have been investigated in pre-clinical and clinical settings [3,4]. Several studies reported DDIs effects in different patients’ populations, especially in the elderly, who are frequently on poly-therapy treatments. Moreover, ADRs lead by DDIs can increase the number and/or duration of hospitalizations and minimize patient’s compliance. In the last few years, many medical tools and pieces of software have been validated to detect DDIs, with the possibility to include the list of patient’s medications and to obtain a real-time profile of possible interactions. In oncology, DDIs are an important issue since patients usually have several comorbidities requiring medication, besides anticancer therapies. In addition, patients often use self-prescribed drugs, herbs or natural compounds for the management of other conditions or side effects [5,6]. Interactions are not unusual for most anticancer drugs, with potential impairment of treatment efficacy. Despite these assumptions, only three retrospective studies have assessed the prevalence of DDIs during cancer treatments [5,6,7]. Among them, two were conducted in patients receiving intravenous cancer treatment, showing that between 27% to 58% of patients had at least one DDI [6,7]. Similar results were found in a multicenter study in patients treated with oral anticancer drugs [5]. These studies have also identified some determinants for the onset of DDIs during anticancer therapies: number of concomitant drugs, use of over-the-counter drugs, type of anticancer treatment and tumor histology [5,6,7]. However, given the retrospective nature of these studies, it is unclear whether DDIs were related to prescription errors or to drug combinations intentionally selected by clinicians. In 2015, Van Leeuwen RW and colleagues conducted a prospective study in cancer patients starting a new oral or intravenous cancer treatment, in order to identify DDIs and obtain more information about potential determinants that could lead to medical intervention. The authors highlighted 603 DDIs in 302 patients, in which 120 DDIs were potentially clinically relevant. Their identification resulted in a medical intervention in 39 patients (13%), while a further intervention was proposed by the clinical pharmacologist in 42 patients (14%) [8].

To date, studies in cancer patients have shown interactions between anticancer treatments and cumarin derivatives, antiepileptic drugs, triazole derivatives, proton pump inhibitors, antiretroviral drugs, antibiotics and folic acid, demonstrating an average DDI rate ranging from 4% to 40% [6,9,10,11]. However, in these studies patients affected by lung cancer were either small in number or not precisely reported (Supplementary Material, Table S1) [5,6,7,8,12]. Only one work evaluated DDIs in patients with thoracic malignancies (non-small-cell lung cancer, pleural mesothelioma, thymic carcinoma, and pulmonary fibrosis) receiving intravenous chemotherapy, identifying in 53 of 300 patients (18%) a potentially clinically relevant DDI [13]. Advanced non-small-cell lung cancer (aNSCLC) harboring activating epidermal growth factor receptor (EGFR) mutation benefits from oral tyrosine-kinase inhibitors (TKIs) such as gefitinib, erlotinib, afatinib and osimertinib. As described in a review by Peters S., gefitinib, erlotinib and afatinib show extensive inter-individual variability in drug absorption. Gefitinib and erlotinib exhibit pH-dependent solubility that also influences their absorption. These drugs undergo extensive hepatic metabolism predominantly by cytochrome P450 (CYP)-dependent enzymes, such as CYP3A4/3A5 and CYP2D6 for gefitinib, CYP3A4/3A5 and CYP1A1/1A2 for erlotinib and CYP3A4/3A5 for osimertinib [14,15] (Table 1). Therefore, potential factors that might be involved in interactions with EGFR-TKIs are drug transporters, CYP enzymes (inhibitors, inducers or substrates) and acid-reducing drugs (H2-receptor antagonists and proton-pump inhibitors). However, data regarding the influence of DDIs in overall survival (OS) and progression-free survival (PFS) from two retrospective clinical trials in this setting are contradictory [16,17].

In order to clarify this topic, we retrospectively investigated the prevalence of DDIs during EGFR-TKIs treatment and the need of a medical intervention using Drug-PIN for the first time. This is a medical tool capable of identifying unfavorable drug interactions by analyzing patients’ medical profiles.

## 2. Materials and Methods

### 2.1. Study Design

We conducted a real world multicenter retrospective data collection with the purpose of evaluating the prevalence of DDIs in aNSCLC patients under EGFR-TKIs. The primary objective was to assess the potential association between the onset of at least one DDI requiring medical intervention and the patient’s baseline clinical features. The secondary objective was to evaluate the possible impact of the clinical features and concomitant therapies taken in the first three months on the efficacy of the treatment in terms of survival and response. This study included consecutive patients with a confirmed diagnosis of aNSCLC in the 24 months prior to the start of the study. The clinical and biological characteristics, and information about cycles and dosage of anticancer therapies, concomitant pharmacological therapies (pharmacotherapy) and ADRs reported, have been extrapolated from the clinical record. The collected data (age, gender, BMI, liver and kidney function and concomitant drugs) have been entered anonymously in the software Drug-PIN. The study was conducted in accordance with the Declaration of Helsinki and the protocol was submitted to the Ethics Committee of the Coordinating Center, Policlinico Umberto I—Sapienza University of Rome and other participating centers.

Ninety-two NSCLC patients were followed at the Policlinico Umberto I of Rome and were enrolled at Istituto Nazionale dei Tumori of Milan between April 2018 and April 2020. The following inclusion criteria were required: (1) patients with histological or cytological diagnosis of non-small-cell lung cancer already treated or candidates to receive treatment with EGFR-TKIs, in any line; (2) age ≥ 18 years; (3) Eastern Cooperative Oncology Group (ECOG) performance status 0–2; (4) informed consent, signed and dated; (5) patients in physical and mental condition to comply with scheduled visits, treatment plan, laboratory tests and other necessary procedures; (6) taking at least 1 concomitant drug before starting anti-tumor treatment. The exclusion criteria were: (1) patients diagnosed with other inadequately controlled conditions that could compromise participation in the study; (2) patients deprived of freedom or under the authority of a legal guardian; (3) patients with a psychological, family, social or geographical condition that could compromise the study protocol.

Toxicities associated with anti-cancer treatment have been evaluated according to the parameters provided by the National Cancer Institute Common Toxicity Criteria, version 5. The survival parameters considered were the objective response rate (ORR), median PFS and median OS. Patients were evaluated with radiological investigations in accordance with clinical practice with a frequency ranging between 12 and 16 weeks. The evaluation of the images was performed using the RECIST criteria (v. 1.1). The ORR was defined as the portion of patients who experienced an objective response (complete or partial response) as the best response to treatment. The PFS was defined as the time from the beginning of treatment to disease progression or death. OS has been defined as the time from the beginning of treatment to death. For PFS and OS, patients without events were considered censored at the time of the last follow-up. Data cut-off period was September 2020.

### 2.2. Concomitant Medications

Each drug taken by the patient at baseline and during the first three months of treatment was considered and collected in categories as shown below:

- gastric acid suppressant, classified according to their indication as no vs. gastritis/gastroesophageal reflux disease (GERD) versus prophylaxis (e.g., to prevent gastritis due to other concomitant medication), no versus H2 antagonists (such as ranitidine) vs. proton-pump inhibitors;
- statins (yes vs. no);
- other lipid-lowering agents (fibrates, ezetimibe and similar) (yes vs. no);
- aspirin (considered as low-dose daily assumption of aspirin for cardiovascular prevention) (yes vs. no);
- anticoagulants (including new oral anticoagulants, low-molecular weight heparin and cumarinic anticoagulant drugs) (yes vs. no);
- NSAIDs, including COX-2 inhibitors (including both chronic and PRN administration) (yes vs. no);
- ACE inhibitors/angiotensin II receptor blockers (ARBs) (yes vs. no), calcium antagonists (yes vs. no), β-blockers (yes vs. no);
- metformin (yes vs. no) and other oral antidiabetics (yes vs. no);
- opioids (yes vs. no);
- antidepressants/antipsychotics (yes vs. no).

### 2.3. Drug-PIN

Drug-PIN^®^ (Personalized Interactions Network) is a software capable of evaluating the pharmacological effectiveness and interactions between drugs. It considers the following data: age, gender, smoking habit, alcohol consumption, laboratory data related to liver and kidney function, pharmacogenomics data and concomitant therapies. Drug-PIN, through semantic analysis algorithms and machine learning, performs a multi-pass analysis and increases the polynomial order of calculation for each element added to the patient’s record. Drug-PIN uses official data from the European Agency of Medicines, FDA, CPIC/Pharm GKB and Open Data. The software provides a score of DDIs based on multiple patients’ drug interactions and also classifies the single drug’s grade of interaction as either low, medium or high. In addition, it calculates a total score that also considers the clinical and laboratory characteristics and genomic data of 110 SNPs of the patient.

### 2.4. Statistical Analysis

Clinical and demographic characteristics, treatment information and types of DDIs are summarized in a descriptive manner. The categorical variables are presented as frequencies and percentages. The continuous variables are presented as mean and standard deviations, medians and interquartile range and 95% confidence interval, depending on the nature of the distribution. The PFS and OS have been evaluated using the Kaplan–Meier method and have been represented graphically. The toxicities of the treatments were evaluated by collecting biochemical parameters and reported symptoms. The collected data were entered in the data collection form and reported in a specific database. Fisher’s exact test was used to evaluate the association between the dichotomous variables. Univariate and multivariate logistic regression models were used to identify variables associated with survival (dependent variable); predictive variables included were: baseline clinical characteristics, concomitant drugs and the total score provided by Drug-PIN. Predictive variables with values of *p* < 0.05 to univariate were included in the multivariate analysis. Based on literature data, it was estimated that DDIs and ADRs are detectable in 18% of thoracic malignancies. The sample was estimated according to available literature data, based on the expected enrollment and the descriptive intent of the study. Considering the number of advanced EGFR mutated NSCLC treated in two years with TKIs (90) in the involved centers, and an expected prevalence of DDIs of 0.054%, assuming a 95% confidence level and an estimated accuracy of +/− 5%, at least 80 patients had to be enrolled, with a minimum median follow-up of 24 months. Statistical analysis was performed using SAS version 9.4, SAS Institute, Cary, NC, USA.

## 3. Results

### 3.1. Patients’ Characteristics

A total of 92 patients were enrolled. The baseline characteristics of patients and data related to concurrent treatments are listed in Table 2. The median age was 68.5 years (range 43–89) with a male/female ratio of 31/61; all patients were taking at least one concomitant drug to the main cancer therapy, of which 14 patients were taking between one and two, and 78 patients three or more concomitant drugs. In total 74 patients had up to two comorbidities and 18 patients had three or more comorbidities requiring concomitant medical therapy. The median PFS and median OS were 16 months (IC 95% range 11–20) and 16 months (IC 95% range 14–23), respectively (Supplementary Material, Figure S1).

### 3.2. Drug–Drug Interactions and Toxicities

Three hundred forty-two DDIs have been identified by Drug-PIN^®^ in the total population and all have been assessed for the need of a medical intervention. Among them, 20 DDIs, occurring in a total of fourteen patients (15%), were considered potentially clinically relevant and in need of medical intervention; 14 of these DDIs (70%) involved the main cancer therapy. The main drugs involved in an interaction with the TKIs used were: selective serotonin reuptake inhibitors—SSRIs (citalopram, escitalopram and fluoxetine), the antipsychotic quetiapine, the antiepileptic carbamazepine, the H2-receptor antagonist ranitidine and the calcium antagonists ivabradine. Sixty-seven patients showed at least one toxicity of any grade, of which seventeen were grade ≥ 3. The median scores provided by the Drug-PIN system of DDIs and total interaction were 73% and 14%, respectively. All potential drug–drug interactions involving antineoplastic therapies are listed in Table S2 (Supplementary Material).

### 3.3. Potential Risks Factors

**Figure 1:**
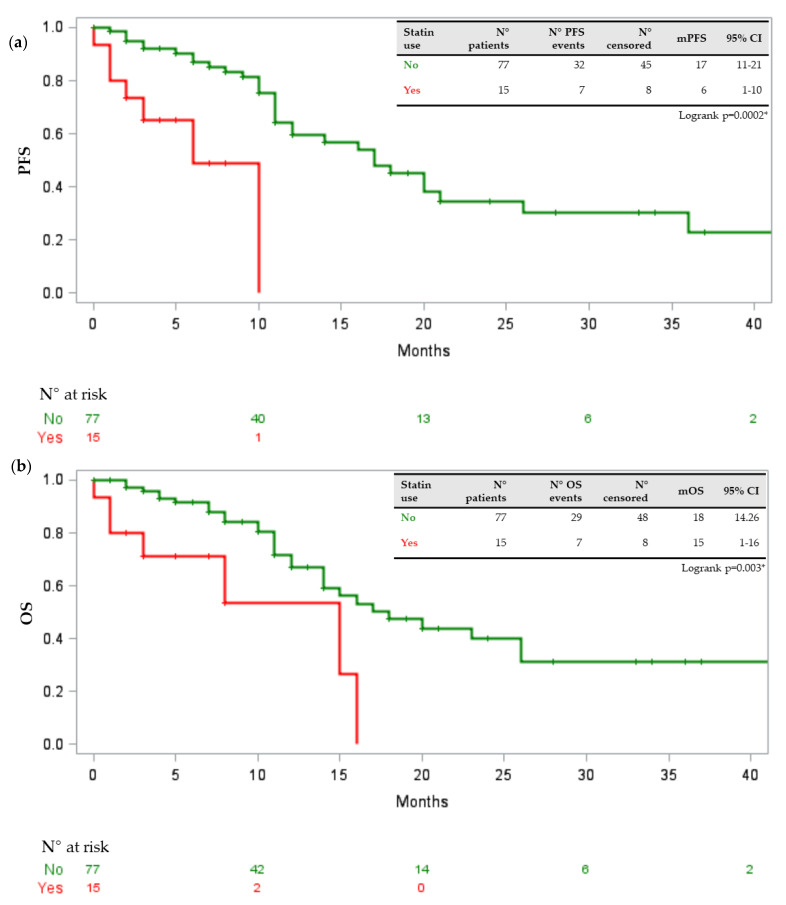
(**a**) Kaplan–Meier curve describing PFS in relation to statins. (**b**) Kaplan–Meier curve describing OS in relation to statins. Legend: PFS progression-free survival; OS overall survival; + censored; * statistically significant.

**Figure 2:**
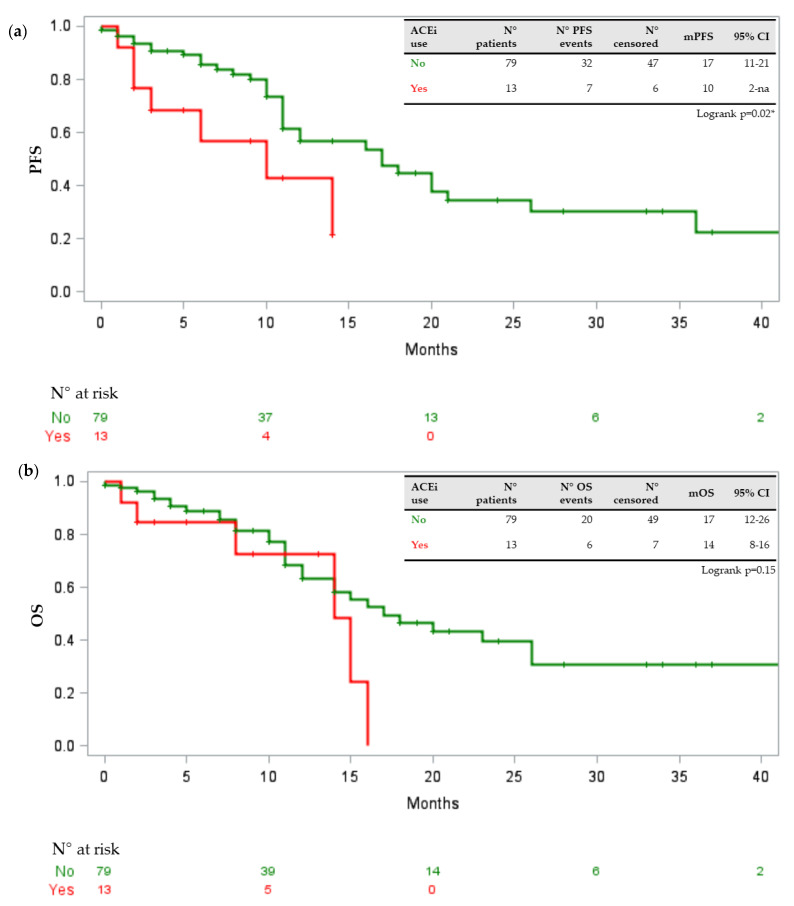
(**a**) Kaplan–Meier curve describing PFS in relation to ACE inhibitors. (**b**) Kaplan–Meier curve describing OS in relation to ACE inhibitors. Legend: PFS progression-free survival; OS overall survival; ACEi ACE inhibitors; + censored; * statistically significant.

**Figure 3:**
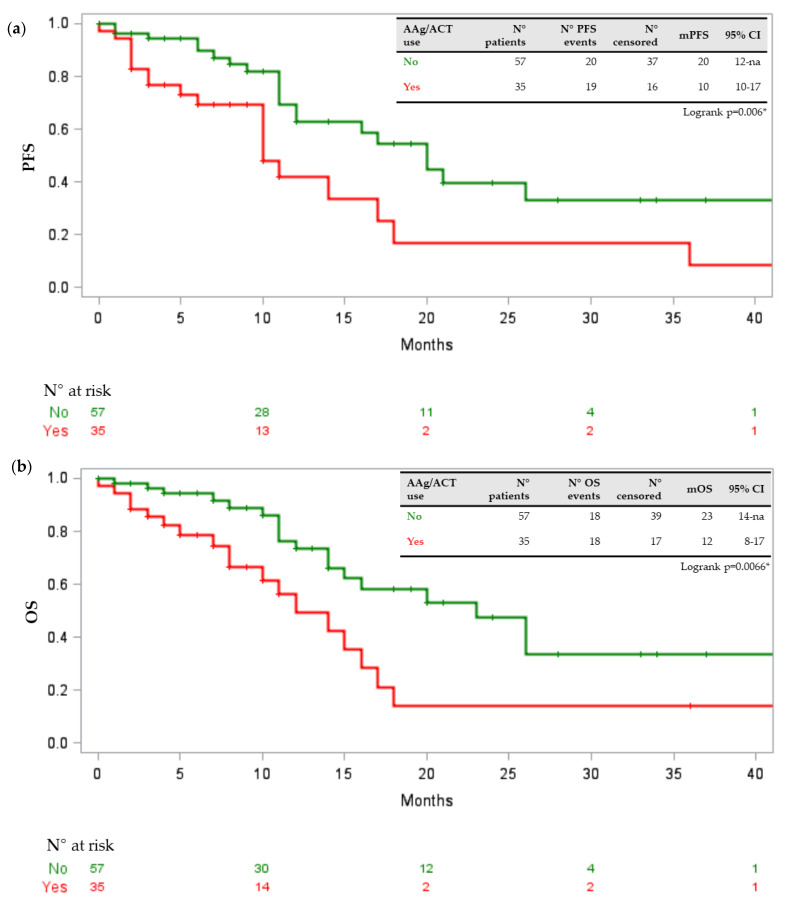
(**a**) Kaplan–Meier curve describing PFS in relation to antiaggregant/anticoagulant drugs. (**b**) Kaplan–Meier curve describing OS in relation to antiaggregant/anticoagulant drugs. Legend: PFS progression-free survival; OS overall survival; AAg antiaggregant; ACT anticoagulant; + censored; * statistically significant.

In our population, age above or equal to 70 years was statistically associated with the presence of a DDI requiring medical intervention with a p-value of 0.04 (odds ratio (OR) 3.594, 95% confidence interval (CI) 1.035–12.47). No association was found with gender, comorbidity number, the kind of TKI (first/second generation versus third generation), number of concomitant drugs, disease burden, ECOG performance status, ORR and onset of toxicity of any grade or severe grade. At the univariate analysis, assessing the relation between baseline clinical features, concomitant drugs and scores provided by Drug-PIN with survival parameters, statistically significant associations between PFS reduction and statin intake (*p* = 0.0002), ACE inhibitors (*p* = 0.002) and antiaggregant/anticoagulant drugs (*p* = 0.006) were identified. Moreover, OS was negatively associated with statin intake (*p* = 0.0030) and the use of antiaggregant/anticoagulant drugs (*p* = 0.0066). Correcting for significant variables found in univariate analysis, only the association between statin intake and PFS was confirmed (*p* = 0.02; HR 0.281, 95%CI 0.096–0.825) at the multivariate analysis stage. Figure 1, Figure 2 and Figure 3 show the Kaplan–Meier curves of PFS and OS in relation to statins, ACE inhibitors, and antiaggregant/anticoagulant drugs.

## 4. Discussion

This is the first study that retrospectively assessed the prevalence of DDIs and the clinical need for medical intervention in patients with advanced NSCLC in treatment with EGFR-TKIs. In total, 20 DDIs were considered as potentially clinically relevant. An incidence of 15% of DDIs in our population is consistent with available literature data [5,6,7]. The main interactions found were in patients treated with the third generation TKI osimertinib, which is also the most prescribed treatment in our cohort. For osimertinib, the most common ADRs (almost all grade 1 or 2) are diarrhea (41%), rash (34%), dry skin (23%) and paronychia (22%). Although not frequent, QT prolongation is one of the known ADRs, occurring in about 4% of patients (almost all grade 1 or 2) [18]. In case of grade 3 QT prolongation, temporary discontinuation of treatment is recommended as there is an increased risk of sudden cardiac death due to ventricular tachycardia or torsade de pointes. The concomitant use of osimertinib with SSRIs such as fluoxetine or citalopram/escitalopram, or with the antipsychotic drug quetiapine or calcium antagonist drugs, may lead to heart rhythm alterations and increase the risk of QTc interval prolongation. This risk increases with the concomitant use of multiple drugs that can prolong QTc and/or with the use of CYP3A4/3A5 inhibitors. Especially antiemetic drugs (e.g., domperidone and 5HT3-antagonists) and some anticancer drugs (tamoxifen, tyrosine-kinase inhibitors and anthracyclines) can elongate the QTc interval [19]. Cardiological monitoring with ECG is recommended 24 to 48 h before and one week after the possible initiation of a concomitant drug that prolongs QTc [20]. Carbamazepine is an inducer of CYP3A4; therefore, its concomitant use may reduce osimertinib blood levels.

The number of comorbidities and the number of concomitant drugs have been previously identified as potential determinants for the onset of a DDI [6,7]. In our study, these parameters are not correlated with increased risk of DDIs. This may have been caused by the fact that only the absolute number of drugs used and comorbidities were correlated in multivariate analyses in previous studies. In patients treated with EGFR-TKIs, age over 70 years was found to be associated with the presence of a DDI requiring medical intervention; these data may reflect the fact that elderly patients are generally affected by multiple comorbidities requiring specific drugs. Furthermore, this population may experience a reduction in renal and/or hepatic function with consequent alteration of pharmacokinetic pathways.

It has been shown that, in cancer patients, increased cholesterol synthesis correlates with a worse prognosis; moreover, cholesterol levels play a role in the development of drug resistance since overexpression of genes involved in its pathway has been observed in tumors refractory to active treatments [19,20,21,22,23,24]. Statins inhibit the synthesis of endogenous cholesterol by acting on the enzyme hydroxymethylglutaryl-CoA reductase, which converts the molecule of 3-hydroxy-3-methylglutaryl-CoA into mevalonic acid, a precursor of cholesterol. They are the most used drugs for the treatment of hypercholesterolemia and have been proven to reduce both morbidity and mortality of cardiovascular events [25,26]. Preclinical studies on lung cancer cell lines have seen how statins induce apoptosis and inhibit tumor growth and angiogenesis [27,28,29]. Recent clinical data have shown how the prolonged use of statins reduces the risk of mortality in patients with lung cancer [30,31]. Hung MS and colleagues have demonstrated, in a cohort of 1707 NSCLC patients treated with gefitinib or erlotinib and concomitant statins, a reduction in the risk of death and a significant increase in PFS and OS [32]. However, the authors used the TKI anti-EGFR response as a surrogate for the mutational status of EGFR. In addition, the use of statins has been shown to overcome resistance to EGFR-TKIs in preclinical and clinical models [33,34]. In particular, Ali et al. demonstrated, in induced EGFR mutated TKI-resistant NSCLC cell lines, a major tumor growth inhibition and reduction in cholesterol levels with the concomitant use of atorvastatin and gefitinib [35]. Cholesterol, as a component of the plasma membrane and lipidic rafts, plays a very important role in regulating the transduction pathway of EGFR intracellular signal [36,37]. In addition, anti-EGFR-TKIs mediate cell death via the intrinsic pathway of apoptosis, therefore high levels of mitochondrial cholesterol in tumor cells may be able to protect against cell death through changes in the permeability of the mitochondrial membrane itself [38,39]. Our study showed that the use of statins was negatively associated with PFS in patients treated with EGFR-TKIs but not with OS; this suggests a negative statin drug interaction with TKIs rather than a worse performance status in patients on statin treatment. Our sample was mostly composed of patients treated with osimertinib; this is a third generation irreversible EGFR-TKI with high sensitivity to both activating EGFR mutations and T790M resistance. Considering that there are no literature data on the effect of statins in patients with NSCLC treated with osismertinib, these results are not yet clear.

The limitations of the study are its retrospective nature, the small sample size, the unavailable enzymatic gene polymorphisms data and no information about over-the-counter medications or any other complementary or alternative therapies that may have affected cancer therapy [40]. However, it constitutes the first attempt to evaluate drug interactions with tyrosine-kinase inhibitors, particularly with osimertinib, using an innovative medical tool.

## 5. Conclusions

There is a need to conduct a prospective double cohort study in which DDIs are identified and corrected in one cohort and identified but not corrected in the other, in order to better explore the effects on the onset of ADRs. Patients with lung cancer are treated in a multidisciplinary manner and there is not always a comprehensive view of all new drugs prescribed. Therefore, the use of a medical tool that documents all of a patient’s drugs, including over-the-counter drugs, and the close collaboration between oncologists and clinical pharmacologists, are necessary to facilitate the identification of the interaction between drugs. The combination of DDIs and individual genetic polymorphisms, called drug–drug–gene interaction (DDGI), is a new fascinating concept of personalized medicine. Drug-PIN^®^ or similar software could be very useful in the new era of personalized medicine to prevent severe adverse events, decrease hospitalizations, and improve survival in cancer patients. Despite being preliminary, the results of this study could help physicians and clinical pharmacologists to be more aware of DDIs in oncology and should lead to closer collaboration to identify and manage DDIs before and during cancer treatment. Further investigations are needed to confirm our data and better investigate the utility of Drug-PIN^®^ in the new era of personalized medicine.

## Figures and Tables

**Table 1:** Cytochrome P450 (CYP450) enzymes involved in the metabolism of EGFRs-TKIs approved for the treatment of patients with NSCLC.

	Metabolyzed by CYP										Can Inhibit	Can Induce
	3A4	3A5	2D6	1A1	1A2	1B1	2C8	2C9	2C19	2E1		
Erlotinib	+++	+++	+	+	++	+	+	+	-	-	CYP3A4 (m)	CYP1A1 CYP1A2
											CYP2C8 (m)	
											CYP1A1 (s)	
Gefitinib	+++	++	+++	++	+	-	-	-	-	-	CYP2C19 (w) CYP2D6 (w)	-
Afatinib	-	-	-	-	-	-	-	-	-	-	-	-
Osimertinib	+++	+++	-	-	-	-	-	-	-	-	-	CYP3A (w)
												

Notes: +++, major metabolic pathway; ++, other significant metabolic pathway; +, minor metabolic pathway; -, no interaction. Abbreviations: w, weak; m, moderate; s, strong.

**Table 2:** Patients’ characteristics. No patients were taking other hypolipidemic therapies or NSAIDs as concomitant therapies.

Study Population	N°	%
**Age**		
<70	50	54
≥70	42	46
**Sex**		
Male	31	34
Female	61	66
**ECOG PS**		
0–1	82	89
≥2	10	11
**BMI (kg/m^2^)**		
Median	22	
	(16.4–36.5)	
**Treatments**		
Gefitinib	6	6
Afatinib	13	14
Osimertinib	73	80
**Disease burden**		
0–2 sites	34	37
3 and more than 3	58	63
* including primitive lesion		
**Concomitant medications**		
Up to 2	14	15
3 and more than 3	78	85
**Comorbidities**		
Up to 2	60	65
3 and up	18	20
**Statins**		
Yes	15	16
No	77	84
**ACE inhibitors**		
Yes	13	14
No	79	86
**Sartans**		
Yes	14	15
No	78	85
**C** **alcium antagonists**		
Yes	10	11
No	82	89
**B-blockers**		
Yes	24	26
No	68	74
**Anticoagulants/aspirin**		
Si	35	38
No	57	62
**Gastric acid suppressant**		
Yes	64	70
No	28	30
**Metformin**		
Yes	7	8
No	85	92
**Insulinotherapy**		
Yes	1	1
No	0	99
**Other oral antidiabetics**		
Yes	3	3
No	89	97
**Antidepressants/antipsychotics**		
Yes	23	25
No	69	75
**Oppioids**		
Yes	18	20
No	74	80

## Data Availability

Data are contained within the article.

## Supplementary Materials

The following are available online at https://www.mdpi.com/article/10.3390/jpm11050424/s1, Figure S1: Kaplan–Meier describing PFS and OD curve in all population, Table S1: Studies that evaluated DDIs in lung cancer patients, Table S2: Identified DDIs involving EGFR tyrosine-kinase inhibitors.

## Abbreviations

DDI	drug-drug interaction
aNSCLC	advanced non-small-cell lung cancer
EGFR	epidermal growth factor receptor
TKIs	tyrosine-kinase inhibitors
NSCLC	non-small cell lung cancer
ADRs	adverse drug reactions
OS	overall survival
PFS	progression free survival
ECOG	Eastern Cooperative Oncology Group
ORR	objective response rate
GERD	gastroesophageal reflux disease
