# Supplementary material for: Evaluation of Drug—Drug Interactions in EGFR-Mutated Non-Small-Cell Lung Cancer Patients during Treatment with Tyrosine-Kinase Inhibitors
Source: J Pers Med. 2021 May 18;11(5):424. doi: 10.3390/jpm11050424 (PMC8157378; doi:10.3390/jpm11050424)

Supplemetary Material

Tables

Table S1 Studies that evaluated DDIs in lung cancer patients.

Study	Treatment	Total patients	Lung cancer patients	Kinf od study
Riechelmann RP et Al. [8]	ev CHT; mABs; OT	405	19 (5,0%)	retrospective
Van Leeuwen et Al [6]	ev CHT; mABs; OT	278	23 (8,3%)	retrospective
Van Leeuwen et Al [5]	os CHT; TKIs; OT	898	28 (3,1%)	retrospective
Van Leeuwen et Al [9]	CHT; mABs; TKIs; OT	121	25 (8,3%) *	prospective
Lopez-Martin C et Al [13]	CHT	75	17 (23%)	prospective
Rompelman FMV et Al [14]	CHT	298	260 (86%)	prospective

Abbreviations: ev, intravenous; os, oral; CHT, chemotherapy; mABs, monoclonal antibodies; OT, hormone therapy; TKIs, tyrosine kinase inhibitors. * Lung cancer patients considered in the group "others".

Table S2 Identified DDIs involving EGFR tyrosine kinase inhibitors.

**DDI total number**	**342**
**DDI requiring medical intervention**	**20**
**DDI involving EGFR TKIs requiring medical intervention**	**14**
**DDI requiring medical intervention**<br>**Yes**<br>**No**	 **14**<br>**78**
**Major DDI involving the main oncological treatment**	**Osimertinib**<br>**+ Escitalopram/Citalopram/Quetiapine/ Carbamazepine/Ivabradina/Fluoxetine**<br><br>**Gefitinib**<br>**+**<br>**Ranitidine**

Supplemetary Material

Figures

Figure S1

a Kaplan-Meir describing PFS curve in all population.

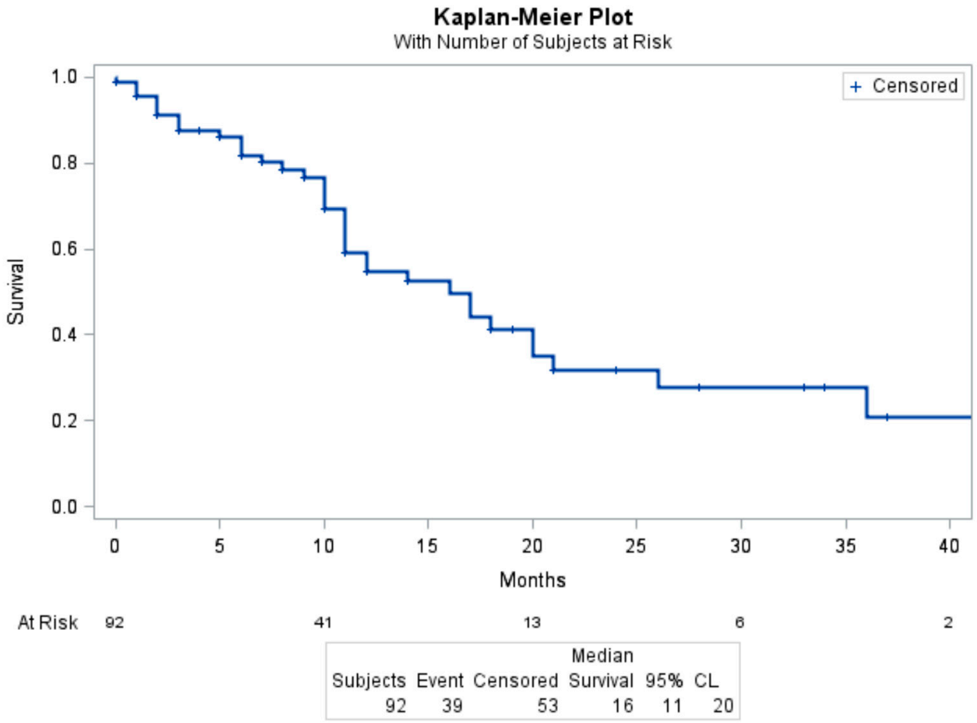

b Kaplan-Meir describing OS curve in all population.

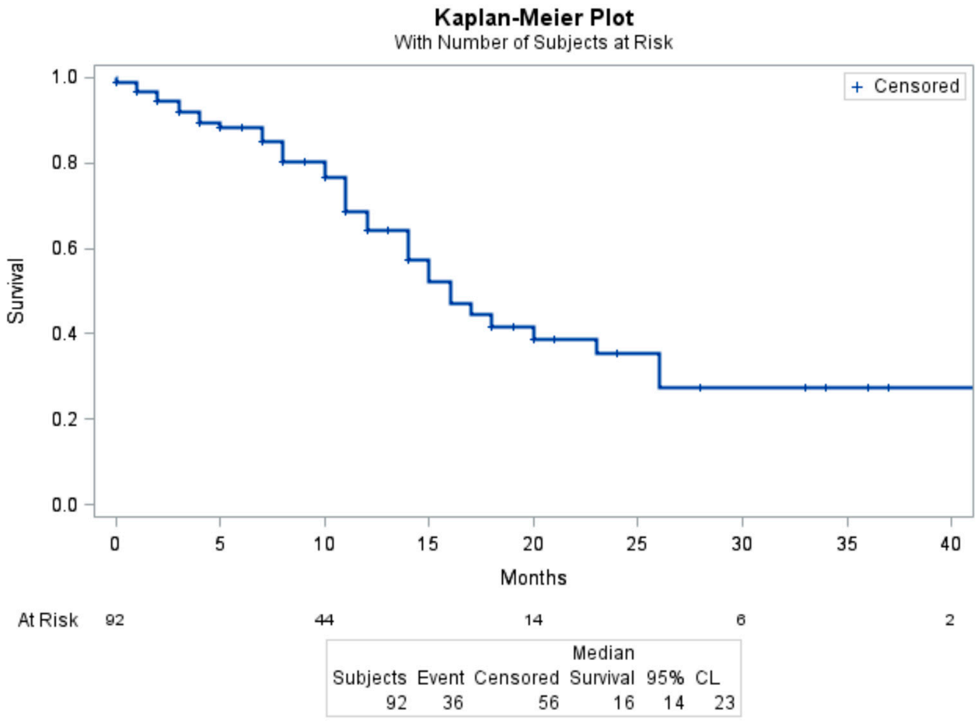

Supplement: Supplementary file 1 [file jpm-11-00424-s001.zip › jpm-1153476-supplementary.pdf]
